# The Ant and the Grasshopper: Does Biased Cognition Compromise Agency in the Case of Delusions and Conspiracy Theories?

**DOI:** 10.1007/s13164-024-00760-x

**Published:** 2024-11-19

**Authors:** Lisa Bortolotti

**Affiliations:** 1 Philosophy Department, University of Birmingham, Edgbaston, Birmingham B15 2TT, UK

## Abstract

This paper starts from an observation of our practices: when people are ascribed delusional beliefs or conspiracy beliefs, they tend to be excluded from shared epistemic projects relevant to the content of their beliefs. What might motivate this exclusion? One possibility is that delusional beliefs and conspiracy beliefs are considered as evidence of irrationality and pathology, and thus endorsing them suggests that one’s epistemic agency is compromised, at least in some contexts. One common argument for the irrational and pathological nature of delusional beliefs and conspiracy beliefs lies in their being the outputs of faulty cognition. There are some areas of overlap in the cognitive antecedents of delusional beliefs and conspiracy beliefs as identified in the empirical literature. For instance, some biases and thinking styles have been correlated either with the adoption of delusional beliefs and conspiracy beliefs, or with the strength of conviction in such beliefs. The paper challenges the claim that, if delusional beliefs and conspiracy beliefs are the outputs of biased cognition, then it is justified to exclude people endorsing delusional beliefs and conspiracy beliefs from epistemic projects relevant to the content of their beliefs. The relevant biases and thinking styles are widespread in the non-clinical population, do not need to be part of a dysfunctional cognitive process, and can be adaptive in some contexts.

## 1 Beliefs with a Delusional Quality

What are delusional beliefs? What are conspiracy beliefs? There are no definitions that are widely accepted and uncontroversial. Some define delusions as false, idiosyncratic, and as denoting abnormality (see e.g., Kiran and Chaudhury 2009), whereas others openly reject falsehood and idiosyncrasy as necessary features of delusions (see e.g., Coltheart 2007). Some define beliefs in conspiracy theories as epistemically irrational—in the sense that the explanation offered by the conspiracy theory is not as evidentially supported as the explanation the conspiracy theory is designed to conflict with (see Ichino and Räikkä 2021). Others challenge the view that conspiracy theories are irrational by default (see Dentith 2016). These disagreements should not stop us from investigating beliefs that are called “delusions” or “conspiracy theories” in lay contexts (Napolitano and Reuter 2023; Pierre 2020). Notwithstanding the obvious heterogeneity of the phenomena, such beliefs may be thought to share some characteristics (a delusional quality): they are attributed to a speaker by an interpreter when they seem to be unshakeable identity beliefs for the speaker but appear as implausible to the interpreter (Bortolotti 2023).

The starting point here is an observation of social interactions in lay contexts. Being ascribed delusional beliefs and conspiracy beliefs often disqualifies a person from collaboration in shared epistemic projects relevant to those beliefs. This may be due to the conviction that delusional beliefs and conspiracy beliefs are irrational and pathological beliefs, outputs of faulty cognition. In this paper I argue that, based on the cognitive antecedents of delusional and conspiratorial beliefs as identified in the empirical literature, this conviction is not justified. Of course, there may be other reasons to think that delusional beliefs and conspiracy beliefs are irrational and pathological, or that collaborations with people who are ascribed such beliefs should not be pursued, but I will not address them here.

Here, I am challenging the idea that collaboration on shared epistemic projects with speakers who are ascribed delusional beliefs or conspiracy beliefs is to be avoided, because the mere presence of such beliefs suggests that speakers’ epistemic agency is compromised, at least with respect to the content of those beliefs. After examining the psychological literature on the biases and thinking styles correlated with the development and persistence of delusional beliefs and conspiracy beliefs, we are left with no good reason to believe that endorsing such beliefs is evidence of compromised agency. This means that the beliefs being outputs of biased cognition does not legitimise the frequent exclusion and pathologisation of those who endorse them.

In Sect. 2, I point out the various attitudes that we can have towards people we interact with, depending on the aims of the interaction. When we aim at coordinating with others, we may need to be able to explain and predict their behaviour in a specific context, and the intentional stance serves us well. That means that we assume they are intentional agents who act on their beliefs, desires, and other intentional states. But when we aim to pursue common epistemic projects with other people, that is, engage in genuine collaboration, we may need to embrace some further assumptions about our collaborators that go beyond the intentional stance. I suggest that the agential stance can capture that.

In Sect. 3, I observe that, when speakers are ascribed delusional beliefs or conspiracy beliefs, such attributions often have the effect of ruling out the adoption of the agential stance towards the speakers, at least with respect to the content of their beliefs. This does not mean that the speakers are no longer recognised as agents, but that genuine epistemic collaboration with them is avoided. The question that interests me is why people are disqualified from engagement in this richer sense when they are ascribed delusional beliefs or conspiracy beliefs. There are many possible answers to that question, but I focus on the idea that delusional beliefs and conspiracy beliefs are regarded as irrational and pathological, that is, as evidence that the person’s agency is compromised in some epistemically relevant respect.

In Sect. 4, I consider some of the factors contributing to our explanations of significant events in critical circumstances: these include data gathering biases (such as jumping to conclusions), psychological needs (such as the need for control, the need for certainty and the need for closure), and biases in the generation and selection of plausible hypotheses (such as the intentionality bias and causality biases). Although such biases and needs are usually more accentuated in the behaviour of people who endorse delusional beliefs or conspiracy beliefs, they are characteristic of the way in which all human agents seek explanations.

In Sect. 5, I start from an Aesop-style fable to illustrate the widespread nature of the biases and needs identified as cognitive antecedents of delusional beliefs and conspiracy beliefs. It is important to acknowledge that delusional beliefs and conspiracy beliefs can be the output of biased cognition. However, identifying some of their antecedents as biases does not justify the assumption that the beliefs are irrational or pathological, and thus does not vindicate the phenomenon of interpreters avoiding collaboration with speakers. People endorsing beliefs with a delusional quality should not be excluded by default from shared epistemic projects.

## 2 Coordination versus Collaboration

When we interact with other systems in the world, we are often interested in explaining and predicting their behaviour. In Daniel Dennett’s account, the intentional stance is one of three stances we can adopt for this purpose. As he says,

“one treats the system whose behavior is to be predicted as a rational agent; one attributes to the system the beliefs and desires it ought to have, given its place in the world and its purpose, and then predicts that it will act to further its goals in the light of its beliefs”. (Dennett 1988, p. 496).

Dennett describes how we attribute beliefs and desires to ourselves and others, arguing that we mostly do so successfully. According to his *intentional stance* theory, we ascribe to a system the beliefs and desires it ought to have and predict the system’s behaviour on the basis of those beliefs and desires.

Now, the intentional stance is *objectifying* to an extent, as the system whose behaviour needs to be explained or predicted is not considered in all its complexities but is attributed intentional states for the specific purposes of the interpreter. That is not necessarily a bad thing. In an online game of chess with an unidentified player, we see the other system merely as a chess player, with a well-defined set of wants—mostly, playing the game and winning. As managers drafting a rota for the shifts of five customer care assistants in a busy city shop, we see the other systems not as complex individuals with a number of potentially conflicting interests and desires, but as employees with a well-defined set of needs—mostly, working a certain number of hours, well distributed in the working week. When we perform specific tasks involving other systems, we need to know only about some of the features of those systems, those that are relevant to our specific task. Similarly, we need to be able to predict how those systems will react to what we do in that specific context but, if all goes smoothly, do not need to know what the systems will do outside of that context. The objectifying nature of the intentional stance is not a problem for those tasks where interpreters are pursuing instances of effective coordination with other systems.

But there are some projects that involve more than coordination. For such projects, interpreters want or need to collaborate with other systems and pursue common epistemic goals. The intentional stance may be a precondition for collaboration because interpreters need to see other systems minimally as intentional if they want to pursue epistemic goals together with them. However, the complexity of interactions leading to successful collaboration requires other assumptions than those captured by the intentional stance. For those interactions, I suggest, interpreters need to adopt an “intentional stance *plus*”, or an *agential stance* (Bortolotti and Murphy-Hollies 2023; Bergen et al. 2022). When adopting the agential stance, interpreters view the target systems as agents who have a valuable perspective on the world, legitimate concerns, a multiplicity of interests and needs that are worth addressing, the capacity to bring about change, and the capacity to participate in decision-making processes based on those interests and needs.

Some of the interpreter’s epistemic goals involve collaborating with speakers whose behaviour is successfully understood and predicted via the intentional stance. This may happen because the target systems have knowledge that the interpreter lacks and that is required for achieving the desired goal. Or it may happen because a decision needs to be made which reflects the needs and values of the speakers as well as those of the interpreter. In a slogan, we move from the intentional stance to the agential stance when we want or have to do things *with* other systems, not just *to* other systems.

For instance, how do we help a younger sibling respond to a bully at school? To devise a successful strategy, we need to take into account the impact it will have on our sibling, and that seems to depend on what their perspectives, needs, and interests are. How do we discuss the merits of a policy or the aesthetic value of an artwork? This sort of discussion involving values requires sharing perspectives and exchanging reasons, navigating areas of agreement and disagreement. So, pursuing epistemic goals together requires more than just explaining and predicting the other system’s behaviour.

## 3 The Withdrawal of the Agential Stance

In some situations interpreters resist the upgrade from the intentional to the agential stance. When an interpreter attributes a delusional or conspiratorial belief to a speaker, something interesting often happens, namely, the intentional stance is adopted but does not evolve into an agential stance. This is one feature that sets delusional beliefs and conspiracy beliefs apart from other beliefs that may be rejected just for being a mistake or a distraction. When people are ascribed delusional beliefs and conspiracy beliefs, they stop being treated as agents worth engaging with in the pursuit of common epistemic goals. They are still agents in the sense that their actions are seen as the effect of their beliefs and desires, and other intentional states, but they are no longer considered as worthy collaborators in shared epistemic projects, at least when such projects are relevant to the content of their delusional beliefs or conspiracy beliefs. So, why does the attribution of beliefs with a delusional quality may allow coordination but compromises genuine collaboration?

It is the first class of the term for a new lecturer, call her Elena. Suppose Elena enters the classroom and starts writing seemingly random numbers on the board. Then she smiles and says to the students: “This is the attendance code for today.” The students see that their lecturer wants to get the admin out of the way, making sure they are not penalised on their attendance record. They immediately attribute beliefs and desires to Elena: she believes this string of numbers is today’s attendance code for the class, she wants them to digit it on their university app so their attendance is recorded accurately. They input the code on the app and Elena can happily start the class. There is nothing in Elena’s behaviour preventing students from adopting the agential stance towards her when the right sort of situation calls for it.

Now let us consider a different version of this story. Evie enters the classroom and starts writing seemingly random numbers on the board. Then she smiles and says to the students: “This is the meaning of the universe. I was told by the KGB last night. I am sharing this with you so you can prepare for what’s to come.” The students are perplexed. They wait to see whether the lecturer is making a practical joke, whether she is acting up to get a reaction from them, but their perplexity turns into incredulous stares when they realise that Evie is perfectly serious. Can they attribute to her the belief that the string of numbers she wrote on the board tells them about the meaning of the universe? Can they attribute to her the desire to tell them what she heard from the KGB the previous night? Can they predict what she will do next based on those beliefs and desires? They probably can make all of those attributions and predictions, but their task as interpreters has become a lot more difficult and uncertain. Students juggle hypotheses in their heads. Maybe their new lecturer is intoxicated and needs a glass of water. Maybe she is having a psychotic break because she forgot to take her medication.

The students can attribute beliefs and desires to Evie in this scenario. There is often a story—which does not need to have a watertight plot or overall coherence—linking the speaker’s current behaviour to their previous experiences and future goals, and a well-informed, curious interpreter may be able to fill the gaps, that is, reconstruct such a story based on the evidence available to them. Not all interpreters are equally knowledgeable and skilled, or curious and patient, and as a result explanations and predictions of Evie’s behaviour may differ. But one consequence of her exchange with the students is that, if they attribute beliefs to her at all, they will attribute to her beliefs with a delusional quality to them. And this is likely to prevent them from engaging with her in instances of genuine collaboration. If she has a perspective on the world, it will not be a valuable one. If she has concerns, these won’t be legitimate. She won’t be the best person to make plans with because she lacks the capacity to make sensible decisions.

When an interpreter recognises a speaker’s belief as delusional, the attribution is not a neutral description of the speaker’s belief but implies some sort of folk-epistemic disapproval (in the words of Wilkinson 2020). The target system is often discredited as a source of reliable information and thus excluded from the pursuit of common epistemic projects. This may be a local effect (that is, students stop trusting their lecturer’s claims about alleged attendance codes) or a more pervasive effect (that is, students stop trusting the lecturer’s claims altogether). The attribution of a belief as a delusion reveals something important about the interpreter’s attitude towards the content of the belief that goes beyond a mere assessment of the belief being false or lacking justification. It has long-term and pervasive effects on the interpreter’s future attitude towards the speaker.

The choice not to engage a person on the subject of their delusions is not surprising when we are thinking about clinical delusions, but it applies to delusional beliefs in lay contexts too, and beliefs in conspiracy theories as well. This exclusion following the endorsement of a conspiracy theory is shown in some experimental work on stigmatized beliefs (Lantian et al. 2018). Let me offer a couple of examples that share the same pattern: when someone claims that the proponent of the opposed view is delusional or is a conspiracy theorist, the next comment is that an exchange with them on the topic of the delusion or the conspiracy theory is going to be unproductive and should be avoided. When the executive director of South Africa’s Presidential Commission on Climate Change called the country’s coal industry “delusional”, he added: “it’s going to be impossible to have a conversation with the industry if we can’t get past the basic denialism” (Reuters, 22 July 2022). Obviously, the word “delusional” is not referring to the symptoms of a psychiatric disorder here, but to the denialism of the industry. Denialism is often the basis for conspiratorial explanations.

After describing Bolsonaro’s anti-vax attitude at the time of the coronavirus as a form of denialism, in a letter to the *Bulletin of National Research Centre*, Machado Silva (2021) says of Bolsonaro:

Lost in daydream, he also stated categorically that he will not take any vaccine, for he mistakenly claims not to need it on the grounds that he would already have been infected by now. Such an attitude could be just a subject for jokes.

A woman describing the difficulties of dealing with relatives who believed conspiracy theories reports:

“Soon after I started challenging my relative’s beliefs, she stopped telling me about the new things she’d learned. I only came to know more about her beliefs through second hand accounts. Perhaps I am at fault here too; I should’ve been more patient and understanding when I was explaining things to her.

But, in reality, it’s difficult for her to hear me out, and it’s difficult for me to hear her out. Both of us think that the other has been brainwashed — I think that she has been brainwashed by conspiracy theorists, and she believes that I have been brainwashed by media sellouts. As you can imagine, it’s an impossible conversation to have.

We still talk a lot — just not about COVID-19, vaccines, or medicine. Those topics are now off-limits”. (Chua 2021).

When a view is considered delusional, a form of denialism, or the endorsement of a conspiracy theory, it is implied that the view should not be taken seriously (“it is a joke”) or engaged with (“the topic is off limits”). The speaker to whom that view is attributed is still treated as an agent in many ways but is no longer a candidate for collaboration and inclusion in common epistemic projects. There are different ways to justify the withdrawal of the agential stance that often follows the attribution of a delusional belief or a conspiracy belief. In the rest of the paper, I shall attempt to defuse one potential justification, that delusional beliefs and conspiracy beliefs are irrational and pathological, and, as outputs of faulty cognition, they are evidence that a speaker’s epistemic agency is compromised.

## 4 Biased Cognition

We jump to conclusions when we make a decision or opt for an explanation of an event without waiting to have sufficient evidence to support that decision or that explanation.

The jumping to conclusions (JTC) bias has traditionally been associated with the formation of persecutory delusions (e.g., Garety et al. 2011), which are the type of delusions most commonly compared to conspiracy beliefs in form and content. In the psychological and clinical literature, there are some controversies surrounding the claim that people with delusions jump to conclusions (see e.g., Huq et al. 1988; Moritz and Woodward 2005; Dudley et al. 2016), partly because there are multiple ways of understanding what JTC precisely involves (Fine et al. 2007) and partly because whether JTC accounts for delusion formation may depend on the type of delusion under examination (Sulik 2023).

That said, aspects of the jumping to conclusions bias, such as hasty reasoning, have been robustly associated with both persecutory delusions and beliefs in conspiracy theories (Sulik 2020; So 2016).^1^ For instance, some have observed that in non-clinical samples, “subjects who displayed the JTC-bias presented a more pronounced belief in conspiracy theories” (Pytlik et al. 2020). In particular, people who gather less information before making a decision seem to endorse conspiracy theories more strongly and to prefer an intuitive as opposed to an analytic thinking style. In the context of conspiracy theories related to coronavirus in Germany, it was found that “higher levels of conspiracy belief endorsement were associated with greater JTC and greater paranoid ideation” (Kuhn et al. 2022).

The presence of overlapping belief formation processes in conspiracy beliefs and delusions may be taken to imply that conspiracy beliefs, just like delusions, are a pathological phenomenon. If conspiracy beliefs are formed via the same mechanisms that are responsible for the formation of delusions, then they may share the pathological status of delusions. However, as it is made very explicit by Carmen Sanchez and David Dunning (2021), jumping to conclusions (or “going with our guts”, as they put it) is not a distinctive mark of dysfunction or pathology. We all tend to jump to conclusions, especially in situations where there are incentives to decide or act promptly. Some of us, depending on personality traits and thinking styles, may have a more pronounced tendency to jump to conclusions.

One key factor that can help avoid the jumping to conclusions bias is *tolerance to uncertainty*. What does this mean? When we jump to conclusions, often we do so because we want an explanation or a solution fast, or we are in a rush to make a decision. Everyone finds uncertainty difficult to manage, but some people find it more distressing than others. It is possible that a reduced tolerance to uncertainty or a stronger need for cognitive closure (NFCC) contribute to jumping to conclusions.

The need for cognitive closure is often considered an aspect of intolerance to uncertainty, and can be defined as “a desire for a quick and unambiguous answer to a question” that “may […] act as a motivational factor that determines successful coping with uncertainty” (as in Czernatowicz-Kukuczka et al. 2014). In other words, the need for closure is an expression of, or a reaction to, intolerance to uncertainty. NFCC may lead to endorsing implausible explanations, but thanks to NFCC, we manage anxiety in the face of a crisis by settling on an explanation that then enables us to make a decision or act promptly.

In a chapter discussing the moral psychology of agents in a pandemic, the behaviour of people during the coronavirus pandemic is taken to exemplify this case (Jefferson and Bortolotti 2023). At some stage, people were asked to make important decisions (e.g., whether to take up the offer of the anti-COVID vaccine) without being able to rely on well-established evidence about the consequences of such decisions (e.g., given the lively debates about the effectiveness and safety of the new vaccine). Intolerance to uncertainty in such a context led some people to take a stance (in favour of or against the vaccine) before conclusive evidence was available.

Greater intolerance to uncertainty is again powerfully associated with delusional and conspiratorial ideation. In some influential accounts, intolerance to uncertainty is considered as a causal factor leading to delusions (Bredemeier at al. 2019) and conspiracy beliefs (Maftei and Holman 2022). It can be thought of as another area of overlap that does not have the mark of a pathological feature of human cognition and decision making. Although some of us have a greater need for closure than others, how pressing it is to solve a critical situation often depends on the context. The more stressful the context is, the stronger the need for closure.

What does it take to increase tolerance to uncertainty and resist hasty decisions? There seem to be two fundamental aspects: one involves the capacity to adapt to change and accept ignorance, and the other involves recognising that we cannot exercise control over everything that matters to us—this is linked to the *need for control* that is often discussed as one of the main psychological needs characterising conspiratorial thinking (see e.g., Douglas et al. 2017). In general, we have a tendency to believe that when something significant happens, it is because someone wanted it to happen (that is the *intentionality bias*). The intentionality bias is associated with schizophrenia-like symptoms in healthy individuals: “the persecutory delusions held by some patients may be explicable in terms of an exaggerated intentionality bias, with these patients being more prone to seeing events that happen to them as intentionally caused” (Moore and Pope 2014).

Is there any evidence of the role of an intentionality bias in conspiratorial thinking? Not all studies find evidence of a correlation between conspiracy beliefs and the intentionality bias as such, but they identify correlations between conspiracy beliefs and either *anthropomorphisation* (overattribution of intentionality not limited to ambiguous actions performed by humans) or *hypersensitive agency detection* (the tendency to attribute intentionality and agency to inanimate objects), which seem to be closely linked to intentionality, but have more specific effects than generalised formulations of the intentionality bias (see e.g. Brotherton & French 2015; Douglas et al. 2015).

In a study where accentuated intentionality bias is detected in schizophrenia, the continuity with non-clinical samples is explicitly advocated:

“[it is important to] consider […] differences in schizophrenia in the context of the “normal” biases shown in subclinical populations, rather than thinking of these biases as “present” in populations with schizophrenia and “absent” from normative populations. For example, most people show some degree of intentionality bias, and doing so may not be an issue. However, problems may arise for those with schizophrenia because they show an amplified bias for intentionality, or because they have trouble controlling this bias” (Buck et al. 2018).

This suggests once more that, if conspiratorial and delusional thinking are characterised by skewed attributions of intentionality, where non-intentional events are interpreted as intended by someone, this is not something that singles them out as processes characterised by a dysfunction, but that places them on a continuum with ordinary thinking. This is a claim about the absence of *a difference in kind* between explanations that are based on versions of the intentionality bias, which does not rule out the presence of *a difference in degree*. Explanations that are based on more accentuated versions of the bias may be more implausible than other explanations. In any case, it is important to observe that excessive attributions of intentionality have significant social as well as cognitive dimensions: for instance, as Karen Douglas and colleagues notice, levels of education play a role in hypersensitive agency detection.

There is another interesting bias that seems to cut across the clinical and non-clinical context, determining how we account for significant events. As psychologist Helena Matute says, *the causality bias*, also known as the illusion of causality, occurs when people believe that there is a causal relationship between events that are actually independent of each other (Matute et al. 2015). Matute explains that identifying causal relationships can be a real challenge: we do not directly observe causality but need to rely on cues to establish causal links between events. Cues include the observation that causes temporally precede effects and that they are relatively close to their effects.

Given the difficulty in establishing causal relationships, it is not surprising that errors are made. In general, “that cognitive biases are not restricted to pathological states and that they often appear in healthy individuals is reinforced by research showing that even experts can fall prey of biases routinely” (Moreno-Fernández et al. 2021). This is true of the causality bias too. Although it is more prominent in delusional thinking and conspiratorial thinking, there is clear continuity in the way people “join the dots” across clinical and non-clinical contexts. One proposal (Balzan et al. 2013) is that delusions emerge when people connect events that have no actual connection—which may be due to causal illusions or overestimations of control.

One key feature of conspiratorial thinking is the rejection of the idea that a significant event could be due to a coincidence, accompanied by the suggestion that there is a causal connection instead, even when such connection seems vague and implausible. As Reine van der Wal and colleagues (2018) claim, “the tendency to draw implausible causal connections between events is a crucial driver of conspiracy thinking” and applies not only to random events and but also to events that conform to an objective pattern.

Although science denial and vulnerability to misinformation are often explained by reference to social factors such as scientific illiteracy, it is productive to explore potential cognitive factors as well such as contingency illusion biases. Perceived causal connections are considered as an important factor in the adoption of conspiracy beliefs in studies on science denialism (Sulik et al. 2020) and on the capacity to distinguish fake news (Saltor et al. 2023). Seeing connections where there are none contributes to the denial of scientific explanations and makes it harder for people to recognise misinformation.

## 5 The Ant and the Grasshopper

Here is an Aesop-style fable.

Grasshopper wakes up one morning to find that his supply of seeds for the winter is gone. He is very distressed. Losing the seeds is a disaster and he faces a difficult winter without the supply. He looks around and sees a trail of seeds leading to Beetle who is sleeping peacefully in the grass.

Grasshopper is certain that Beetle stole the seeds, and tells his friend Ant about it. Ant is not so sure that Beetle is responsible for eating the seeds. She thinks Grasshopper is jumping to conclusions and he is too eager to blame Beetle for what happened.

Ant notices that seeds are scattered on the ground, and that fallen leaves and broken branches can be found all over the garden. Considering the evidence available to her, Ant suggests that a strong wind must have scattered the seeds during the night, made the leaves fall from the trees, and caused some branches to snap.^2^

We do not know whether Beetle ate the seeds or whether it was the wind that scattered them, but independent of which explanation is the correct one, we may recognise in Grasshopper some of the characteristics of someone who has paranoia or a tendency to prefer theories involving a conspiracy to other types of explanations.

Grasshopper faces a distressing and unexpected event, the disappearance of his supply of seeds, and seeks an explanation for the event without considering all the evidence that is relevant and available to him. Filling the gaps in the story and imagining how it might further develop, we expect that Grasshopper will hang on to his explanation. It is of some comfort to him to be able to identify a villain—someone responsible for his misfortunes—and, even if Ant’s explanation sounds quite plausible, he does not want to admit that he may have been wrong.

There are similarities and differences in how Ant and Grasshopper gather evidence once they realise that the seeds are no longer there. Both are concerned about the event, and both seek an explanation. They do their own research, and they come up with an explanation that is plausible to them. Grasshopper arrives at his conclusion by following a trail of seeds that points to Beetle, and comes to believe that Beetle stole and ate the seeds.

If pressed to support his theory, Grasshopper would probably draw attention to the fact that Beetle is sleeping soundly, just as someone would after a good meal, instead of looking for food. Grasshopper identifies and infers causal links between a number of facts that he is able to observe: the seeds are gone, there is a trail of seeds leading to Beetle, Beetle is sleeping. Grasshopper feels the pull of causality and joins the dots: if the seeds are gone and the trail of seeds leads to Beetle, then Beetle must have taken them. If Beetle is fast asleep instead of being up and about, looking for food, well maybe he already has a very full tummy.

Ant is not in a rush to come to an explanation of the missing seeds, taking time to consider the available evidence before assessing Grasshopper’s theory. She tolerates uncertainty better than Grasshopper in the circumstances and is also more sympathetic to an explanation that attributes the missing seeds to natural causes and not to the evil intentions of an agent.

Grasshopper’s preferred explanation is a good example of the work of the biases and psychological needs we reviewed in the previous section but is not as implausible as some instances of delusional beliefs and conspiracy beliefs. If some key factors contributing to how agents arrive at delusional beliefs and conspiracy beliefs can be captured by considering how Grasshopper attempts to solve the mystery of the missing seeds, this suggests that such factors are not something that makes delusional beliefs and conspiracy beliefs irrational or pathological by default.

The biases that are implicated in the formation of delusional beliefs and conspiracy beliefs are also features of everyday cognition and agency that surface, at least to some extent, in many attempts to make sense of the physical and social world around us. They are general features of human cognition and agency that are undesirable and costly in specific contexts or for specific purposes but could be desirable and beneficial in other contexts or for different purposes. In a relatively safe society, we aspire to be as conscientious and cool-headed as Ant and avoid blaming other agents unnecessarily. So, jumping to conclusions is bad and carefully reviewing the evidence available to us is good. But in a context where there is an active and imminent threat, or people compete for limited resources, it may be more prudent to act fast and avoid trusting competitors. Hypervigilance can be adaptive in some contexts and become a hindrance in other contexts (Palafox-Harris 2024).

In this paper I limited my analysis to those cognitive biases that are commonly considered in the empirical literature as cognitive antecedents of delusional beliefs and conspiracy beliefs (most notably, in the influential work of Joseph Pierre and Karen Douglas). I have not reviewed other possible factors that might suggest the presence of a dysfunctional belief-formation process. Limited to the evidence I considered here, there does not seem to be any reason to think that delusional beliefs and conspiracy beliefs are by default irrational or pathological.

First, the cognitive antecedents I considered are described and conceptualised as biases. The very term “bias” (as opposed to “deficit”) indicates a context-dependent behavioural tendency triggered by environmental cues whose consequences may vary based on the nature of the task at hand. Second, the relevant cognitive antecedents are widespread and not unique to clinical samples, that is, to people who have been diagnosed with a mental disorder. It is possible that delusional beliefs and conspiracy beliefs are to be regarded as pathological because some processes in their formation are dysfunctional and lead to pathological behaviour, but the cognitive antecedents I have considered do not seem on their own to be dysfunctional or to lead to pathological behaviour. Third, building on the previous two points, reliance on the cognitive antecedents identified in the literature is not a sign of irrationality by default or independent of context. As pointed out when discussing the Aesop-like fable, the use of the strategies and biases I have reviewed can turn out to be psychologically and epistemically beneficial and preferable to the alternatives depending on the situation in which agents find themselves and the epistemic goals they intend to pursue.

It is significant that delusional beliefs and conspiracy beliefs emerge in situations that call for the exercise of epistemic agency: the search for an explanation, the need to make a decision, the attempt to solve a problem. Such beliefs may not offer the best available explanations, may lead to suboptimal decisions, and may offer solutions that are ineffective, or that are rejected by others, but they seem to be produced in the exercise of agency just as their non-delusional and non-conspiratorial counterparts would be. This conflicts with the claim that, when speakers are ascribed a belief with a delusional quality, the belief signals a failure of agency: “He doesn’t know what he’s saying”; “She’s insane”; “I really don’t understand how they can genuinely believe this”; “They must have been brainwashed”. Implications often drawn from attributions of delusional beliefs and conspiracy beliefs are that the speaker who endorses those beliefs is not an agent with whom it is advisable or productive to pursue shared epistemic projects. The interpreter’s language *pathologizes*: there must be something wrong with speakers who behave in those ways and, although coordination and manipulation may still be possible, though arduous, genuine epistemic collaboration and a rich engagement are ruled out.

The assumption I am challenging here has important consequences for our mutual interactions: when speakers are ascribed beliefs with a delusional quality, interpreters may think that it is pointless to attempt to understand the speakers’ perspective (because speakers don’t have a perspective that is worth engaging with, at least in the context of the delusional belief or the conspiracy belief) or exchange reasons with the speakers (because speakers are thought to be irresponsive to evidence or arguments, at least in the context of the delusional belief or the conspiracy belief). So, there is no productive conversation with the health guru who believes that COVID-19 was a hoax because germ transmission is a myth of modern medicine, or with Trump supporters who claim that his inauguration in 2017 was better attended than Obama’s in 2009. The perspectives of agents who are ascribed delusional beliefs and conspiracy beliefs are partially or totally written off.

A closer look at delusional beliefs and conspiracy beliefs reveals that it is by no means straight-forward to find principled ways to demarcate them from other beliefs, and this might explain why defining them seems to be so hopeless. The discussion of what happens in the garden to Ant and Grasshopper is just an attempt to resist simplistic ways of justifying pathologizing and agency-defying interpretations of delusional beliefs and conspiracy beliefs. There are no likely cognitive antecedents of delusions and conspiracy beliefs that are not also likely antecedents of beliefs full-stop. Lay conceptions of conspiratorial and delusional thinking as pathological and agency-defying by default are untenable if the factors contributing to delusional beliefs and conspiracy beliefs are continuous with the factors contributing to non-delusional and non-conspiratorial beliefs. And such continuity has been remarked and investigated in influential accounts of delusion-like experiences and cognitions in non-clinical contexts (see for instance, van Os 2003).

What can we do to avoid excluding and pathologizing people who are ascribed beliefs with a delusional quality to them? There are already excellent suggestions about how to improve communication by being open-minded, receptive, and non-confrontational (Douglas et al. 2024). Let’s start by accepting that interpretation can be hard, but that some difficulties in understanding other speakers’ perspectives do not necessarily mean that adopting the agential stance will be unproductive or pointless. Let’s also recognise that some of the biases and thinking styles that are at least partially responsible for delusional beliefs and conspiracy beliefs operate in those situations where epistemic agency is exercised, and are more prominent in situations that are unexpected and stressful for the agents involved. Finally, let’s accept that those biases and thinking styles can hinder epistemic goals in some contexts and promote them in others. Ant’s approach to solving the mystery of the missing seeds may be more analytic, reflective, open-minded, and collaborative than Grasshopper’s, but it may be Grasshopper’s suspiciousness and quick associative thinking that will help the garden animals overcome the next crisis.

## Data Availability

No datasets were generated or analysed during the current study.

## Declarations

1 In terms of the range of application of JTC, it has been recently argued that it is not a transdiagnostic phenomenon (e.g., it is not relevant to depression), but in at least some of its formulations (i.e., hasty reasoning) it is significant in schizophrenia and other psychotic disorders. JTC is also reliably associated with conspiracy beliefs – this has been shown in a number of recent studies. Some authors such as Sulik (2023) do not deny the strong correlation between delusions and jumping to conclusions, but suggest possible factors that can explain or mediate that correlation. Whether those factors invalidate the connection between jumping to conclusions and delusions may depend on how they are operationalised.

2 “The Ant and the Grasshopper” is an animated video available at The Philosophy Garden: https://youtu.be/_RfC_Zwf6oI.

## References

[R1] Balzan RP, Delfabbro PH, Galletly CA, Woodward TS (2013). Illusory correlations and control across the psychosis continuum: The contribution of hypersalient evidence-hypothesis matches. Journal of Nervous Mental Disorders.

[R2] Bergen C, Bortolotti L, Tallent K (2022). Communication in youth mental health clinical encounters: Introducing the agential stance. Theory & Psychology.

[R3] Bortolotti L (2023). Why Delusions Matter.

[R4] Bortolotti L, Murphy-Hollies K (2023). Why we should be curious about each other. Philosophies.

[R5] Bredemeier K, McCole K, Luther L, Beck AT, Grant PM (2019). Reliability and validity of a brief version of the intolerance of uncertainty scale in outpatients with psychosis. Journal of Psychopathology and Behavioral Assessment.

[R6] Brotherton R, French CC (2015). Intention seekers: Conspiracist ideation and biased attributions of intentionality. Plos One.

[R7] Buck B, Hester NR, Pinkham A, Harvey PD, Jarskog LF, Penn DL (2018). The bias toward intentionality in schizophrenia: Automaticity, context, and relationships to symptoms and functioning. Journal of Abnormal Psychology.

[R8] Chua AD (2021). My loved ones are caught in a web of conspiracy theories — here’s what it’s like. The Varsity.

[R9] Coltheart M (2007). Cognitive neuropsychiatry and delusional belief (the 33rd Sir Frederick Bartlett lecture). The Quarterly Journal of Experimental Psychology.

[R10] Czernatowicz-Kukuczka A, Jaśko K, Kossowska M (2014). Need for closure and dealing with uncertainty in decision making context: The role of the behavioral inhibition system and working memory capacity. Personality and Individual Differences.

[R11] Dennett D (1988). Precis of *The Intentional Stance*. Behavioral and Brain Sciences.

[R12] Dentith MRX (2016). When inferring to a conspiracy might be the best explanation. Social Epistemology.

[R13] Douglas KM, Sutton RM, Callan MJ, Dawtry RJ, Harvey AJ (2016). Someone is pulling the strings: Hypersensitive agency detection and belief in conspiracy theories. Thinking & Reasoning.

[R14] Douglas KM, Sutton RM, Cichocka A (2017). The psychology of conspiracy theories. Current Directions in Psychological Science.

[R15] Douglas KM, Sutton RM, Biddlestone M (2024). Engaging with conspiracy believers. Review of Philosophy and Psychology.

[R16] Dudley R, Taylor P, Wickham S, Hutton P (2016). Psychosis, delusions and the jumping to conclusions reasoning bias: A systematic review and meta-analysis. Schizophrenia Bulletin.

[R17] Fine C, Gardner M, Craigie J, Gold I (2007). Hopping, skipping or jumping to conclusions? Clarifying the role of the JTC bias in delusions. Cognitive Neuropsychiatry.

[R18] Garety P, Freeman D, Jolley S, Ross K, Waller H, Dunn G (2011). Jumping to conclusions: The psychology of delusional reasoning. Advances in Psychiatric Treatment.

[R19] Huq SF, Garety PA, Hemsley DR (1988). Probabilistic judgements in deluded and non-deluded subjects. Quarterly Journal of Experimental Psychology Section A.

[R20] Ichino A, Räikkä J (2021). Non-doxastic conspiracy theories. Argumenta.

[R21] Kiran C, Chaudhury S (2009). Understanding delusions. Industrial Psychiatry Journal.

[R22] Kuhn S, Lieb R, Freeman D, Andreou C, Zander-Schellenberg T (2022). Coronavirus conspiracy beliefs in the german-speaking general population: Endorsement rates and links to reasoning biases and paranoia. Psychological Medicine.

[R23] Lantian A, Muller D, Nurra C, Klein O, Berjot S, Pantazi M (2018). Stigmatized beliefs: Conspiracy theories, anticipated negative evaluation of the self, and fear of social exclusion. Eur J Soc Psychol.

[R24] Maftei A, Holman A-C (2022). Beliefs in conspiracy theories, intolerance of uncertainty, and moral disengagement during the coronavirus crisis. Ethics & Behavior.

[R25] Matute H, Blanco F, Yarritu I, Díaz-Lago M, Vadillo MA, Barberia I (2015). Illusions of causality: How they bias our everyday thinking and how they could be reduced. Frontiers in Psychology.

[R26] Moore JW, Pope A (2014). The intentionality bias and schizotypy. Quarterly Journal of Experimental Psychology (2006).

[R27] Moreno-Fernández MM, Blanco F, Matute H (2021). The tendency to stop collecting information is linked to illusions of causality. Science Reports.

[R28] Moritz S, Woodward TS (2005). Jumping to conclusions in delusional and non-delusional schizophrenic patients. British Journal of Clinical Psychology.

[R29] Napolitano MG, Reuter K (2023). What is a Conspiracy Theory?. Erkenntnis.

[R30] Palafox-Harris E, Sullivan-Bissett E (2024). The Routledge Handbook of the philosophy of delusions.

[R31] Pierre JM (2020). Mistrust and misinformation: A two-component, socioepistemic model of belief in conspiracy theories. Journal of Social and Political Psychology.

[R32] Pytlik N, Soll D, Mehl S (2020). Thinking preferences and conspiracy belief: Intuitive thinking and the jumping to conclusions-Bias as a basis for the belief in conspiracy theories. Frontiers in Psychiatry.

[R33] Reuters (2022). Coal industry is ‘delusional’, South Africa climate change official says.

[R34] Saltor J, Barberia I, Rodríguez-Ferreiro J (2023). Thinking disposition, thinking style, and susceptibility to causal illusion predict fake news discriminability. Applied Cognitive Psychology.

[R35] Sanchez C, Dunning D (2021). People who Jump to conclusions Show other kinds of thinking errors. Scientific American.

[R36] Silva HM (2021). The danger of denialism: Lessons from the Brazilian pandemic. Bulletin of the National Research Centre.

[R37] So SH, Siu NY, Wong HL, Chan W, Garety PA (2016). Jumping to conclusions’ data-gathering bias in psychosis and other psychiatric disorders - two meta-analyses of comparisons between patients and healthy individuals. Clinical Psychology Review.

[R38] Sulik J, Ross RM, McKay RT (2020). The contingency illusion bias as a potential driver of science denial.

[R39] Sulik J, Ross RM, Balzan R, McKay R (2023). Delusion-like beliefs and data quality: Are classic cognitive biases artifacts of carelessness?. Journal of Psychopathology and Clinical Science.

[R40] The Philosophy Garden (2023). The Ant and the Grasshopper.

[R41] van der Wal RC, Sutton RM, Lange J, Braga JPN (2018). Suspicious binds: Conspiracy thinking and tenuous perceptions of causal connections between co-occurring and spuriously correlated events. European Journal of Social Psychology.

[R42] van Os J (2003). Is there a continuum of psychotic experiences in the general population?. Epidemiologia E Psichiatria Sociale.

[R43] Wilkinson S (2020). Expressivism about delusion attribution. European Journal of Analytic Philosophy.

